# Supplementary material for: Using Normalized Carcinoembryonic Antigen and Carbohydrate Antigen 19 to Predict and Monitor the Efficacy of Neoadjuvant Chemotherapy in Locally Advanced Gastric Cancer
Source: Int J Mol Sci. 2023 Jul 29;24(15):12192. doi: 10.3390/ijms241512192 (PMC10418931; doi:10.3390/ijms241512192)
Supplement: Supplementary file 1 [file ijms-24-12192-s001.zip › Supplementary Figure 1.pdf]

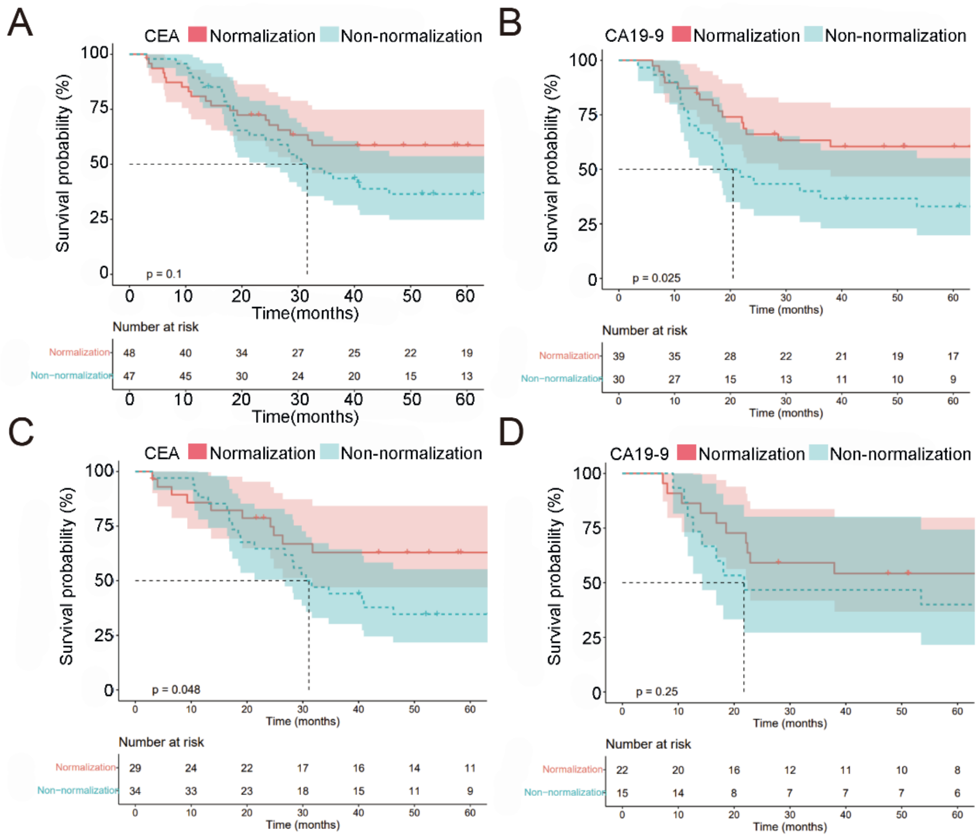

Figure S1. Overall survival by changes at CEA or CA19-9 status. (A) Overall survival among patients with normalization and non-normalization of CEA following NACT, regardless of the status of CA19-9. (B) Overall survival among patients with normalization and non-normalization of CA19-9 following NACT, regardless of the status of CEA. (C) Overall survival among patients with normalization and non-normalization of CEA following NACT, while the status of CA19-9 is negative. (D) Overall survival among patients with normalization and non-normalization of CA19-9 following NACT, while the status of CEA is negative. NACT: neoadjuvant chemotherapy.
