# Supplementary material for: Using Normalized Carcinoembryonic Antigen and Carbohydrate Antigen 19 to Predict and Monitor the Efficacy of Neoadjuvant Chemotherapy in Locally Advanced Gastric Cancer
Source: Int J Mol Sci. 2023 Jul 29;24(15):12192. doi: 10.3390/ijms241512192 (PMC10418931; doi:10.3390/ijms241512192)
Supplement: Supplementary file 1 [file ijms-24-12192-s001.zip › Supplementary table 1.pdf]

Table S1. Data of clinicopathological information in patients with or without postoperative CEA/CA19-9 normalization

Characteristics	Normalization (n=73)	Non-normalization (n=59)	p value
Age in years, median (range)	61 (36-82)	62 (45-86)	0.916
Gender			0.741
Male, n (%)	59 (80.8)	49 (83.1)	
Female, n (%)	14 (19.2)	10 (16.9)	
BMI (median)			0.220
< median, n (%)	33 (45.2)	33 (55.9)	
> median, n (%)	40 (54.8)	26 (44.1)	
Location			0.827
Upper, n (%)	30 (41.1)	25 (42.4)	
Middle, n (%)	11 (15.1)	8 (13.6)	
Lower, n (%)	31 (42.5)	26 (44.1)	
Total, n (%)	1 (1.4)	0 (0.0)	
Diameter			0.054
< median, n (%)	42 (57.5)	24 (40.7)	
> median, n (%)	31 (42.5)	35 (59.3)	
Differentiation			0.629
Differential	34 (46.6)	25 (42.4)	
Undifferential	39 (53.4)	34 (57.6)	
cT stage			0.621
T1-T2, n (%)	1 (1.4)	2 (3.4)	
T3, n (%)	26 (35.6)	18 (30.5)	
T4, n (%)	46 (64.0)	39 (66.1)	
cN stage			0.464
N0, n (%)	8 (11.0)	4 (6.8)	
N1, n (%)	24 (32.9)	14 (23.7)	
N2, n (%)	28 (38.4)	27 (45.8)	
N3, n (%)	13 (17.8)	14 (23.7)	
cTNM stage			0.892
II, n (%)	9 (12.3)	6 (10.2)	
III, n (%)	60 (82.2)	49 (83.1)	
IV, n (%)	4 (5.5)	4 (6.8)	

BMI, body mass index.
