# Supplementary material for: Using Normalized Carcinoembryonic Antigen and Carbohydrate Antigen 19 to Predict and Monitor the Efficacy of Neoadjuvant Chemotherapy in Locally Advanced Gastric Cancer
Source: Int J Mol Sci. 2023 Jul 29;24(15):12192. doi: 10.3390/ijms241512192 (PMC10418931; doi:10.3390/ijms241512192)
Supplement: Supplementary file 1 [file ijms-24-12192-s001.zip › Supplementary table 2.pdf]

Table S2. Proportional hazards model for normalization in CEA or/and CA19–9 following NACT (n = 132)

	Univariable			Multivariable		
	HR	95% CI	p Value	HR	95% CI	p Value
Age (ref: ≤median)	1.02	0.99-1.05	0.079	1.03	1.00-1.05	0.036*
Gender (ref: female)	1.13	0.63-2.04	0.674	-	-	-
BMI (ref: ≤median)	1.00	0.99-1.01	0.222	-	-	-
Location (ref: Lower)	1.05	0.82-1.36	0.695	-	-	-
Differentiation (ref:Undifferential)	0.65	0.41-1.04	0.073	0.64	0.40-1.04	0.069
ypT (ref: T1-2)	1.16	0.93-1.43	0.186	-	-	-
ypN (ref: N0)	1.17	0.97-1.42	0.102	-	-	-
ypTNM stage (ref: II stage)	1.30	0.97-1.75	0.075	1.18	0.87-1.58	0.287
TRG (ref: non-MPR)	1.30	0.99-1.70	0.052	1.18	0.89-1.57	0.260
Normalized CEA/CA19-9 (ref: non-normalized)	0.52	0.32-0.84	0.007**	0.59	0.36-0.97	0.039*

BMI, body mass index; TRG, tumor regression grading; MPR, major pathological response; NACT, neoadjuvant chemotherapy. *$p < 0.05$, **$p < 0.01$.
