# Supplementary material for: Using Normalized Carcinoembryonic Antigen and Carbohydrate Antigen 19 to Predict and Monitor the Efficacy of Neoadjuvant Chemotherapy in Locally Advanced Gastric Cancer
Source: Int J Mol Sci. 2023 Jul 29;24(15):12192. doi: 10.3390/ijms241512192 (PMC10418931; doi:10.3390/ijms241512192)
Supplement: Supplementary file 1 [file ijms-24-12192-s001.zip › Supplementary table 3.pdf]

**Table S3. Serum tumor biomarkers of treatment response to neoadjuvant therapy in gastric cancer patients**

| Study | Biomarker | Biomarker status in responders | NACT regimen | PR definition | PMID |
|---|---|---|---|---|---|
| Li et al. | CEA | No significant association was detected | FLEEOX | pCR | 29670115 |
| Chen et al. | CEA<br>CA19-9<br>CA125<br>CA153<br>AFP | Higher CEA level was associated with higher rate of pCR. | mFLOT, FOLFOX, XELOX, SOX | pCR | 32476803 |
| Ohe et al. | CEA<br>CA19-9<br>CA125<br>AFP | No significant association was detected | mDCS | JCGC (2-3) | 32110104 |
| Sun et al. | CEA<br>CA19-9<br>CA724<br>CA125 | High pretreatment CEA levels (>50 ng/ml) may predict clinical disease progression after NACT. The decrease of tumor markers CEA, CA72-4, and CA125 was significant after NACT. | FOLFOX | CR+PR+SD | 25543664 |
| Our study | CEA<br>CA19-9 | Normalization of CEA/CA19-9 is the strongest predictive marker for treatment response of NACT in LAGC | XELOX, SOX, mFOLFOX7 | pCR + MPR | - |

**Abbreviations:** CEA, carcinoembryonic antigen; CA-125, cancer antigen 125; CA 19–9, cancer antigen 19–9; CA-153, cancer antigen 153; AFP, alfa-fetoprotein; FLEEOX, fluorouracil/leucovorin/etoposide/oxaliplatin/epirubicin; FOLFOX, leucovorin/fluorouracil/oxaliplatin; XELOX, capecitabine/oxaliplatin; MFLOT, fluorouracil/leucovorin/oxaliplatin/docetaxel; SOX, S-1/oxaliplatin; mDCS, docetaxel/cisplatin/S-1; NACT, neoadjuvant chemotherapy; pR, pathological response; pCR, pathological complete response; JCGC, Japanese Classification of Gastric Cancer;

*P-values are presented only for values < 0.05.
